# Using eDNA for mammal inventories still needs naturalist expertise, a meta‐analysis

**DOI:** 10.1002/ece3.10788

**Published:** 2023-12-06

**Authors:** Pauline Van Leeuwen, Johan Michaux

**Affiliations:** ^1^ Conservation Genetics Laboratory University of Liège Liège Belgium

**Keywords:** assessment, biodiversity, eDNA, environmental DNA, iDNA, mammals, meta‐analysis, survey

## Abstract

DNA from the environment (eDNA) has been increasingly used as a new tool to conduct biodiversity assessment. Because of its noninvasive and less time‐consuming nature, many studies of recent years solely rely on this information to establish a species inventory. eDNA metabarcoding has been shown to be an efficient method in aquatic ecosystems, especially for fish. However, detection efficiency is not clear for mammals. Using the existing literature, we conducted a meta‐analysis to investigate if eDNA metabarcoding allows greater detection success compared to conventional surveys (such as field surveys, camera traps, etc.). Although only 28 articles were retrieved, showing the lack of comparative studies, still representing more than 900 taxa detected, we found that detection success was method dependent, but most importantly varies on the taxonomy of the targeted taxa. eDNA metabarcoding performed poorly for bats compared to the traditional mist nests. However, strong detection overlaps were found between conventional surveys and eDNA for large‐bodied mammals such as ungulates, primates, and carnivores. Overall, we argue that using both molecular and field approaches can complement each other and can maximize the most accurate biodiversity assessment and there is much room for metabarcoding optimization to reach their full potential compared to traditional surveys.

## Graphical abstract

We compared conventionnal surveys methods with eDNA for mammal biodiversity inventories. Although more taxa were detected using eDNA, we found low overlap detection between methods, arguing for the use of both methods at this time to maximize the accuracy of biodiversity assessment.

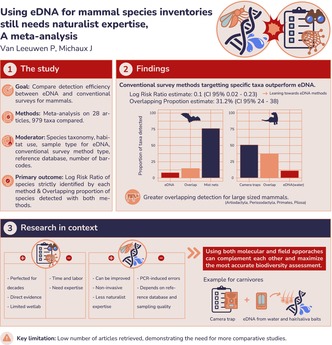

## 1 INTRODUCTION

Mammals are one of the taxa undergoing drastic declines through fast‐paced anthropogenic changes in the landscape, many of them being secluded in protected areas, with extreme conservation status (Benítez‐López et al., 2019; Hill et al., 2020; Pacifici et al., 2020). Hence, we strongly need rapid, cost‐effective, noninvasive biodiversity assessments to monitor the variation of animal communities across changing environments. To conduct such assessments, especially for mammals, conventional methods such as trapping through cages, nets, or camera, as well as field surveys, are conducted (Table 1). Those methods provide direct observations and identifications, as well as information on the studied population (reproductive status, sex, potential disease status of each individual). Such surveys have been deployed and improved over multiple decades to capture a maximum of individuals, with specificities according to each targeted taxon.

**TABLE 1:** Rapid description of common traditional methods for mammalian surveys.

Method	Description
Camera traps	Disposition of a set of camera over multiple locations that record according to movement, day and night. Species identification by a research team based on morphological and behavioral elements from footage.
Field surveys and transects	Visual observation on the field location on a grid and transect unit. Considering the living characteristics of terrestrial species, direct observation and traces (caves, excrement, footprints, etc.) were checked and investigated while moving. Usually conducted during the day (unlikely to detect nocturnal animals).
Field signs	Footprints and scats at sampling locations (or along transects) collected on each occasion (daily).
Areal counts	Helicopter‐based, with trained observers and a data recorder.
Mist netting	Mist nets positioned across potential flight paths of bats along a transects. Set up from duck and were monitored continuously until midnight.
Cage traps	Grid composed of transects with trap stations of two cages with strict intervals. Cage traps baited and checked daily.
Pitfall traps	Approx. 100 m pitfall line of plastic buckets, spaced 10 m apart. Drift fences, consisting of a continuous barrier running the total length of each line, made of strips of hardware clear polyethylene clipped to vertical stakes hammered into the ground.

However, environmental DNA—eDNA—has emerged as a new tool to conduct biodiversity assessment over the last two decades. In this study, we will only focus on eDNA metabarcoding coined by Taberlet et al. (2012) as high‐throughput multi‐species detection using DNA extracted from environmental samples based on PCR and NGS technologies, intentionally excluding single‐species specific detection assay via qPCR, for example, that do not fall into broad mammalian inventory assessments. On a broader scale, eDNA metabarcoding is being used to characterize past and present biodiversity patterns (Zinger et al., 2019), to understand trophic interactions and diet preferences (Galan et al., 2018) and to monitor ecosystem health and dynamics (Evrard et al., 2019). In the case of vertebrates, many different substrates have been used to establish species inventories and can be grouped into three classes. First, the eDNA group, using substrates from the animal's environment such as water (seawater, freshwater, Ficetola et al., 2008; Foote et al., 2012), soil (Andersen et al., 2012; Taberlet et al., 2012), sediments (McDonald et al., 2023; Ryan et al., 2022), and air through filters (Garrett et al., 2023; Lynggaard et al., 2022). Another type of substrate can be considered eDNA traps, as they allow DNA concentration due to their intrinsic properties, such as feces (Van Der Heyde et al., 2021; Walker et al., 2019, hair (Croose et al., 2023; Lee et al., 2016), saliva bait (Nichols et al., 2015; Piaggio et al., 2019); saltlicks (Ishige et al., 2017), vegetation (Allen et al., 2023; Van Der Heyde et al., 2021) and even spider webs (Gregorič et al., 2022). Finally, a third group of DNA originates from invertebrates—iDNA—that are ectoparasites of the targeted taxa and blood/fecal meals are used as DNA sources (Calvignac‐Spencer et al., 2013). Among them are leeches (Hanya et al., 2019; Schnell et al., 2012), flies (Fernandes et al., 2023; Schubert et al., 2015), mosquitoes (Danabalan et al., 2023; Massey et al., 2022), and dung beetles (Drinkwater et al., 2021).

While at first used as a complementary method to conventional surveys, eDNA metabarcoding has become a main stand‐alone technique to estimate species richness on a defined location, because this method presents multiple benefits compared to conventional surveys (Thomsen & Willerslev, 2015; Ruppert et al., 2019). Briefly, sampling collection needs less manpower, taxonomic expertise, and time (linked to costs) to be implemented compared to traditional methods. Collection standardization is also possible over multiple habitat types due to the limited constraint for sample collection. eDNA metabarcoding also implies being less dependent on weather conditions or seasons for substrate access compared to traditional methods that are more dependent on logistic issues, and is completely noninvasive towards the targeted species, as it can also provide high sensitivity, especially for species with cryptic lifestyles. However, due to the PCR‐based nature of eDNA metabarcoding, this method still represents a number of pitfalls and challenges (Beng & Corlett,  2020; Coissac et al., 2012; Taberlet et al., 2012; Thomsen & Willerslev, 2015). The main disadvantages are lack of laboratory access by field scientists in many countries, DNA degradation, persistence and inhibition due to its origin, state, transport and fate in the environment (Barnes & Turner, 2015); PCR and sequencing errors, as well as human‐induced contamination from the field and the wet lab; the accuracy of identification being highly biased by differing quality of reference databases, and finally the lack of information on specimens (age, living or dead, hybrid, sex).

To date, it has been shown that eDNA from water is particularly efficient for aquatic ecosystems, especially amphibians, fishes and freshwater mussels (Carvalho et al., 2021; Keck et al., 2022; Svenningsen et al., 2022) due to the release of DNA through mucus secretion or free gamete and larvaes and their circulation in freshwater (Barnes & Turner, 2015). When it comes to mammals, few studies to date looked at the global detection success for eDNA metabarcoding compared to conventional methods for species richness assessment. Our goal was thus to explore the literature for comparison between eDNA metabarcoding and traditional survey methods and to distinguish which methods allow more taxa detection depending on method types and more particularly according to which terrestrial mammalian taxa. Because the presence of shed DNA is based on physiology, behavior, and ecology of the targeted species (Seeber & Epp, 2022), we expected that eDNA would be less effective for the detection of small mammals and Chiroptera compared to cage trapping and mist nets. We argue that the amount of DNA shed by those taxa would be in lesser amount compared to large ungulates and carnivores. Although bat DNA captured through air filters seems a promising strategy, its utilization is still at the infancy level and studies did not meet our selection criteria (Garrett et al., 2023; Johnson et al., 2023). Moreover, we hypothesized that eDNA from soil was less efficient than originating from freshwater due to the patchy distribution of mammals on land (Seeber & Epp, 2022). To verify that, we retrieved articles where a direct comparison in the same location was done between eDNA metabarcoding and conventional methods, going up to the species level, for mammals, and conducted a meta‐analysis based on detection ratios and overlaps between methods.

## 2 METHODS

### 2.1 Data extraction

**FIGURE 1:**
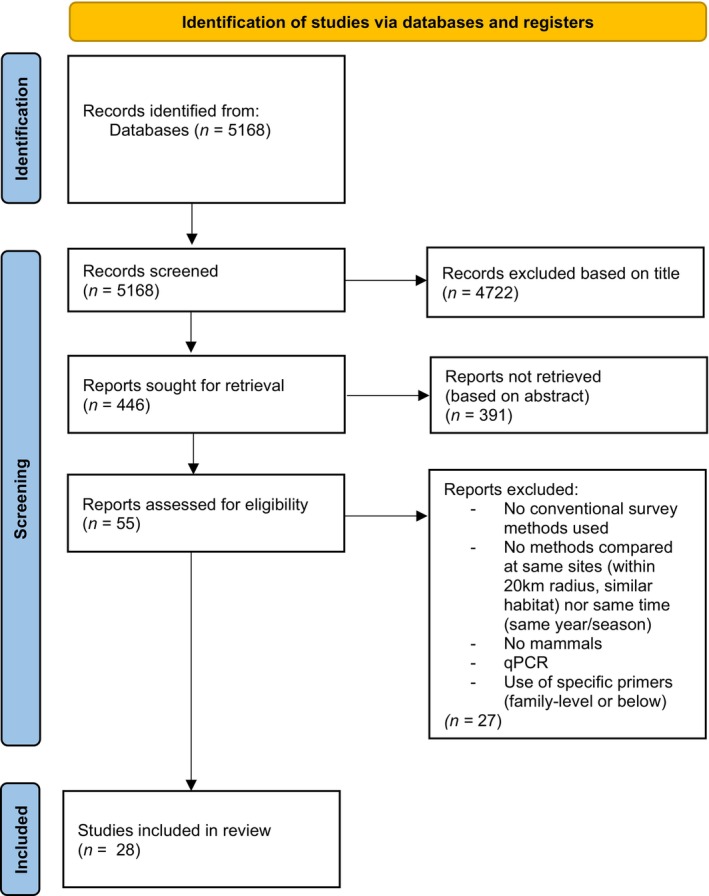
PRISMA flow diagram.

A literature search was conducted from 2005 to 2023 using Google Scholar, Pudmed, Scopus, Science Direct databases as well as in depth search on Environmental DNA and Metabarcoding and Metagenomics journals. Search terms included “(metabarcod* OR "Environmental DNA" OR edna OR "ingested DNA" OR idna) AND (vertebrate OR mammal) AND (traditional OR conventional OR survey OR inventory)”. Papers (*n* = 5168) were manually assessed for relevance based on the abstract to ascertain whether the study met the criteria listed in Figure 1. The following data were extracted from each accepted article: article metadata, sampling information (geographical information, taxa detected, sampling size) and methodological details about methods (length of survey, markers, technology). When extracting data for species richness, data were retrieved as followed when a study compared two methods: the number of species only detected by eDNA, the number of species only detected by a traditional method, and the number of species detected by both methods. The values were separated by taxonomical order, although identification ranged from family to species level individually. In the same way, if traditional survey methods only targeted a reduced set of taxa (bats for mist netting, traps for small mammals), no comparison was included with eDNA barcoding for other taxa when multiple methods were involved within a publication. Additionally, information about the taxa's habitat uses (arboreal, terrestrial, subterranean, aquatic, aerial) and taxonomy were verified and documented using Wilson and Mittermeier (2009).

### 2.2 Meta‐analysis of data

Following Keck et al. (2022) and Carvahlo et al. (2021), we used the log‐ratio risk (LRR) as the log of (ai/n1i)/(ci/n2i), where ai and ci are the number of taxa not detected by eDNA/iDNA or conventional methods, respectively; and n1i and n2i the sample size of each of these two groups (Viechtbauer, 2010). The LRR value is above one when traditional methods detected more taxa than DNA barcoding, below one when DNA barcoding detected more taxa than traditional methods and equals one when both methods are equal. To study the proportion of overlap (PO) between methods, proportion of taxa in common for all sites between eDNA and conventional methods was estimated as a ratio of taxa detected overlapping to the total number of taxa for each publication with double arcsine transformation.

Data were grouped for the statistical analysis based on each moderator investigated and by publication. Therefore, if a study evaluated multiple methods, a single study could yield multiple groups to be included in the meta‐analysis. All data analysis was conducted in R v.4.0.3 (R Core Team, 2018). An overall test for heterogeneity was conducted to inspect publication bias, as well as a funnel and Egger's test for funnel asymmetry. The Cochran's chi‐squared test (*Q*‐test), tau^2^, and Higgin's index (*I* ^2^) were used to measure heterogeneities in the overall dataset and for group analyses. *I* ^2^ estimates greater than 75% were considered high heterogeneities (Wang, 2018).

Random effects model estimations with restricted maximum likelihood method were used to explain the heterogeneity in effect sizes in different moderators using the *metafor* package (V3.0‐2, Viechtbauer, 2010). The following moderators were inspected separately: taxa‐related moderator (taxonomy, habitat use) and survey method‐related moderators (sample type for eDNA, type of conventional method, reference database choice, and number of barcodes). Within study variation was assumed to be different between moderators. Each moderator was evaluated separately in univariate models, while the interaction between animal taxonomy and type of methods were being tested simultaneously in meta‐regression models.

## 3 RESULTS

### 3.1 Literature search

**FIGURE 2:**
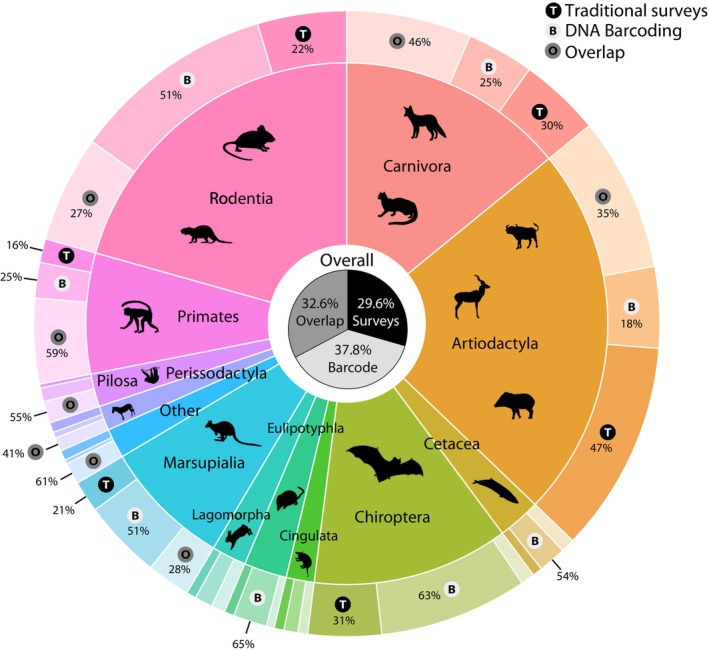
Detection proportion by taxonomic orders according to methods.

A total of 5168 articles were identified within all databases from January 2005 to September 2023 (Figure 1). Articles were retrieved from 2022, 2021, 2020, 2019, 2018, 2017, and 2016, with a maximum of five articles for 2020. A total of 14 mammalian orders composed of 979 taxa were detected overall within the 28 articles retrieved (Appendix S1). The most detected orders (23% of the dataset) were carnivores (*n* = 229/979), followed by rodents (21%, *n* = 202), Artiodactyla (14%, *n* = 138), bats (12%, *n* = 119), marsupials (8%, *n* = 78), and primates (7%, *n* = 73, Figure 2). A majority of the animals were sampled in South America (39%), followed by East Asia (14%), Africa (14%), Europe (13%), and North America (12%). In the same way, 53% of mammals detected were reported to be terrestrial, 13% arboreal, 12% aerial, and 11% semi‐arboreal while semiaquatic, marine and subterranean represented less than 10% in the total dataset. Most of the dataset compared traditional surveys with eDNA coming from water samples (50.4%) and iDNA from flies, leeches, and mosquitoes (35.3%), while few involved water sediments (7%) soil (5%) and tree hollow samples (2.3%). 68% of the dataset included comparison between eDNA/iDNA against camera traps, but other conventional methods such as mist netting (5%), field surveys and transects (18%), cage trapping (3.5%), as well as pitfall traps, areal counts and trawl surveys for marine species (<3%) were also investigated. Studies used the genetic markers 12S, 16S, CO1 and cytochrome b for eDNA/iDNA barcoding, 70% of taxa investigated involved a single genetic marker, two markers for 20% and three for 6% of the dataset, all through an Illumina sequencing platform.

### 3.2 Overall heterogeneity assessment and publication bias

**FIGURE 3:**
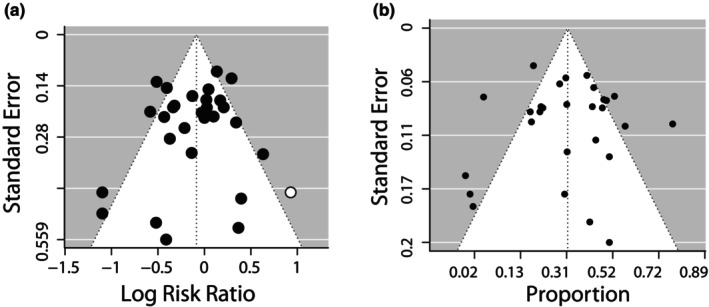
Funnel plots for overall models of LRR and OP.

Heterogeneities in effect sizes were identified and quantified in the entire dataset for both LRR and overlap proportion (OP). The overall estimate in LRR was −0.1026 (95% CI = −0.228; 0.023) while OP overall estimate was 31.23% (95% CI = 24–38%). Moderate to high overall heterogeneities were observed (LRR: Tau^2^ = 0.058; SE = 0.029; *I* ^2^ = 59.14%; OP: Tau^2^ = 0. 0.029; SE = 0.011; *I* ^2^ = 79.9%), with significant variation in LRR and OP between studies (LRR: *Q* = 70.29, *p* < .0001; OP: *Q* = 117, *p* < .0001). In the analysis of publication bias, nonsignificant asymmetries of the funnel plot were noted for LRR and OP among all mammals' detection (Figure 3; LRR: Egger's test: *z* = −1.0447, *p* = .2962; OP: *t* = 0.2243, *p* = .8243).

### 3.3 Moderator analysis

#### 3.3.1 LRR – Taxa only detected by each method

**FIGURE 4:**
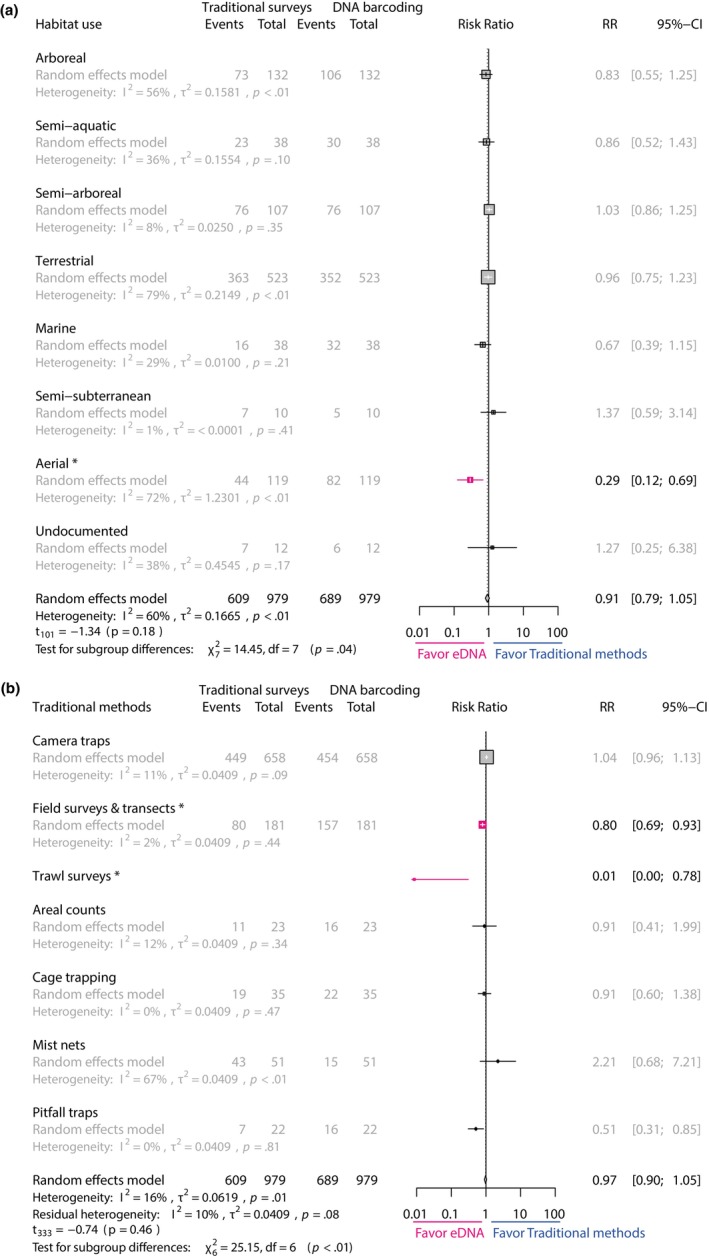
Forest plot for LRR moderator analysis for (a) animal habitat use and (b) type of traditional survey method.

When considering taxonomy of animals detected at the order level, moderator differences for estimates variation in LRR were statistically significant (*Q* = 36, *p* = .0003) with moderate but significant heterogeneity (*I* ^2^ = 48.1%, *Q* = 158, *p* < .0001). Higher LRR was observed when comparing traditional methods compared to eDNA for carnivore detection, meaning more detection through traditional methods (Table 2A). Lower LRR is also observed for aerial animals compared to others (Table 2A, Figure 4a). The type of sample for DNA barcoding had no significant impact on LRR variation within the dataset (*I* ^2^ = 85.6%, *Q* = 1.87, *p* = .74). According to the model, type of traditional method influenced LRR, with greater detection using eDNA compared to transects and trawl surveys (*Q* = 15.4, *p* = .017, Table 2A, Figure 4b). The use of different reference databases for metabarcoding had nonsignificant impact on LRR estimates (*Q* = 3.87, *p* = .276), while lower LRR estimates are observed when studies used only one barcode despite great within group heterogeneity (*Q* = 10.3, *p* = .006).

**TABLE 2:** Moderator analysis results.

Moderator	Subgroup	*k*	RR	95% CI	Tau^2^	Tau	*Q*	*I* ^2^ (%)	*p*‐Value random model
(A) Log risk ratio									
Taxa	Artiodactyla	23	1.0099	[0.8182; 1.2467]	0.0950	0.3082	37.43	41.2	.9233
	**Carnivora**	**26**	**1.3758**	**[1.1346; 1.6681]**	**0.0476**	**0.2183**	**39.57**	**36.8**	**.0022**
	Cetacea	5	0.7241	[0.2050; 2.5582]	0.1822	0.4269	8.74	54.2	.5168
	Cingulata	5	0.7464	[0.3996; 1.3942]	<0.0001	0.0026	3.35	0.0	.2635
	**Chiroptera**	**13**	**0.2916**	**[0.1241; 0.6851]**	**1.2301**	**1.1091**	**43.63**	**72.5**	**.0084**
	Eulipotyphla	10	0.6901	[0.3879; 1.2275]	<0.0001	0.0019	10.93	17.6	.1791
	Lagomorpha	13	0.9269	[0.6081; 1.4128]	<0.0001	0.0015	11.90	0.0	.7016
	Marsupialia	7	0.6506	[0.2873; 1.4733]	0.2558	0.5058	16.71	64.1	.2456
	Perissodactyla	10	0.9957	[0.6701; 1.4794]	0	0	4.98	0.0	.9808
	Pilosa	5	0.7827	[0.4732; 1.2947]	<0.0001	0.0016	4.53	11.6	.2479
	Primates	11	0.9917	[0.8476; 1.1603]	<0.0001	0.0012	12.22	18.2	.9082
	Rodentia	21	0.7030	[0.4803; 1.0289]	0.3861	0.6213	64.25	68.9	.0679
	Other	10	1.2739	[0.9283; 1.7480]	0	0	4.97	0.0	.1175
Habitat use	Arboreal	13	0.8317	[0.5530; 1.2510]	0.1581	0.3976	27.54	56.4	.3447
	Semi‐aquatic	12	0.8598	[0.5170; 1.4300]	0.1554	0.3942	17.32	36.5	.5268
	Semi‐arboreal	22	1.0348	[0.8565; 1.2502]	0.0250	0.1582	22.81	7.9	.7103
	Terrestrial	23	0.9612	[0.7487; 1.2339]	0.2149	0.4635	106.27	79.3	.7452
	Marine	7	0.6720	[0.3936; 1.1474]	0.0100	0.1000	8.44	28.9	.1189
	Semi‐subterranean	7	1.3676	[0.5950; 3.1437]	<0.0001	0.0015	6.07	1.2	.3928
	**Aerial**	**13**	**0.2916**	**[0.1241; 0.6851]**	**1.2301**	**1.1091**	**43.63**	**72.5**	**.0084**
	Unknown	5	1.2671	[0.2517; 6.3802]	0.4545	0.6742	6.43	37.8	.7051
Sample type for DNA barcoding	Invertebrates	8	0.9808	[0.6519; 1.4757]	0.1926	0.4388	58.70	88.1	.9139
	Water	15	0.7584	[0.5189; 1.1083]	0.2667	0.5165	98.14	85.7	.1402
	Soil	2	0.9590	[0.0010; 906.7766]	0.5361	0.7322	12.69	92.1	.9507
	Water/Sediment	3	1.1288	[0.1341; 9.4990]	0.6236	0.7897	13.98	85.7	.8294
	Log debris	1	0.7500	[0.5350; 1.0514]	–	–	0.00	–	.0951
Traditional method compared	Camera traps	21	0.9832	[0.7741; 1.2488]	0.1955	0.4422	120.15	83.4	.8829
	**Field surveys & transects**	**10**	**0.5978**	**[0.4025; 0.8878]**	**0.1140**	**0.3377**	**25.20**	**64.3**	**.0209**
	**Trawl surveys**	**1**	**0.0526**	**[0.0035; 0.7815]**	–	–	**0.00**	–	**.0421**
	Areal counts	1	0.6875	[0.4148; 1.1395]	–	–	0.00	–	.4723
	Mist netting	2	0.5898	[0.0000; 11647.3804]	0.9738	0.9868	4.47	77.6	.7435
	Cage trapping	2	2.0856	[0.0003; 16469.2182]	0.8725	0.9341	7.88	87.3	.4616
	Pitfall traps	1	0.4375	[0.2254; 0.8491]	–	–	0.00	–	.1438
Reference database choice	Custom	7	0.9835	[0.5540; 1.7459]	0.3087	0.5556	37.30	83.9	.9458
	Genbank	12	0.8897	[0.6010; 1.3170]	0.1651	0.4063	62.26	82.3	.5254
	EMBL	8	0.8034	[0.4832; 1.3359]	0.2899	0.5384	68.17	89.7	.3426
	BOLD	1	0.5517	[0.3552; 0.8569]	–	–	0.00	–	.0081
Number of barcodes used	1	18	0.7825	[0.5982; 1.0235]	0.2372	0.4871	148.64	88.6	.0707
	2	8	0.9841	[0.4837; 2.0024]	0.2505	0.5005	28.51	75.5	.959
	3	2	1.2245	[0.5693; 2.6341]	0	0	0.49	0.0	.1841

Moderator	Subgroup	*k*	OP	95% CI	Tau^2^	Tau	*Q*	*I* ^2^ (%)	*p*‐Value random model
(B) Overlapping proportion									
Taxa	**Artiodactyla**	**23**	**0.4923**	**[0.2874; 0.6981]**	**0.0807**	**0.2841**	**61.29**	**64.1**	**<.0001**
	Carnivora	26	0.3127	[0.2225; 0.4087]	0.0115	0.1071	34.82	28.2	.0645
	Cetacea	5	0.1533	[0.0000; 0.6613]	0.0740	0.2721	10.97	63.5	.0626
	Cingulata	5	0.2428	[0.0219; 0.5471]	0	0	2.05	0.0	.2261
	**Chiroptera**	**13**	**0.0000**	**[0.0000; 0.0107]**	**0.0071**	**0.0844**	**8.12**	**0.0**	**<.0001**
	**Eulipotyphla**	**10**	**0.0634**	**[0.0000; 0.2921]**	**<0.0001**	**0.0013**	**8.52**	**0.0**	**.0092**
	Lagomorpha	13	0.2593	[0.0091; 0.6201]	0.0494	0.2222	16.04	25.2	.2684
	**Marsupialia**	**7**	**0.2067**	**[0.0012; 0.5427]**	**0.0593**	**0.2436**	**20.91**	**71.3**	**.0262**
	Pilosa	5	0.5641	[0.1760; 0.9198]	<0.0001	0.0001	4.28	6.5	.6116
	Perissodactyla	10	0.4889	[0.0562; 0.9305]	0.0737	0.2714	13.87	35.1	.9791
	Primates	11	0.5149	[0.2414; 0.7849]	0.0673	0.2594	27.04	63.0	.6060
	**Rodentia**	**21**	**0.2510**	**[0.1561; 0.3560]**	**0.0147**	**0.1214**	**32.30**	**38.1**	**.0162**
	Other	10	0.6573	[0.3024; 0.9493]	0	0	7.99	0.0	.3601
Habitat use	Arboreal	13	0.2920	[0.1233; 0.4858]	0.0358	0.1892	28.79	58.3	.4064
	Semi‐aquatic	12	0.3895	[0.1110; 0.7003]	0.0412	0.2030	17.80	38.2	.0606
	Semi‐arboreal	22	0.4493	[0.2649; 0.6389]	0.0282	0.1678	33.61	37.5	.1332
	Terrestrial	23	0.3582	[0.2682; 0.4528]	0.0283	0.1681	72.33	69.6	.4795
	Marine	7	0.1435	[0.0000; 0.4804]	0.0546	0.2336	13.35	55.1	.4850
	Semi‐subterranean	7	0.1127	[0.0000; 0.5385]	0	0	4.50	0.0	.0748
	**Aerial**	**13**	**0.0000**	**[0.0000; 0.0107]**	**0.0071**	**0.0844**	**8.12**	**0.0**	**.0115**
	**Unknown**	**5**	**0.0231**	**[0.0000; 0.2478]**	**0**	**0**	**1.50**	**0.0**	**.0218**
Sample type for DNA barcoding	Invertebrates	8	0.3460	[0.1635; 0.5534]	0.0501	0.2238	53.60	86.9	.4547
	Water	15	0.2755	[0.1682; 0.3954]	0.0260	0.1613	49.92	72.0	.5737
	**Soil**	**2**	**0.4197**	**[0.3580; 0.4825]**	**0**	**0**	**0.00**	**0.0**	**<.0001**
	Water/Sediment	3	0.2834	[0.0198; 0.6629]	0.0109	0.1046	3.56	43.8	.9882
	Log debris	1	0.5217	[0.3151; 0.7248]	–	–	0.00	–	.3214
Traditional method compared	**Camera traps**	**20**	**0.3545**	**[0.2686; 0.4449]**	**0.0251**	**0.1583**	**68.94**	**72.4**	**.0224**
	Field surveys & transects	10	0.2641	[0.1322; 0.4166]	0.0154	0.1239	16.75	46.3	.5660
	Trawl surveys	1	0.0000	[0.0000; 0.1827]	–	–	0.00	–	.3346
	Areal counts	1	0.1739	[0.0415; 0.3600]	–	–	0.00	–	.9792
	Mist netting	2	0.1587	[0.0426; 0.3146]	0	0	0.02	0.0	.5923
	Cage trapping	2	0.0764	[0.0000; 1.0000]	0.0340	0.1844	3.58	72.1	.9792
	Pitfall traps	1	0.0455	[0.0000; 0.1849]	–	–	0.00	–	.4709
Reference database choice	**Custom**	**7**	**0.4168**	**[0.2171; 0.6303]**	**0.0387**	**0.1968**	**28.77**	**79.1**	**.0232**
	Genbank	12	0.2848	[0.1611; 0.4245]	0.0222	0.1489	30.31	63.7	.0805
	EMBL	8	0.3188	[0.2104; 0.4375]	0.0144	0.1200	27.36	74.4	.0634
	BOLD	1	0.0465	[0.0008; 0.1351]	–	–	0.00	–	.1897
Number of barcodes used	1	18	0.2871	[0.2127; 0.3671]	0.0176	0.1327	59.79	71.6	.1042
	2	8	0.2602	[0.0785; 0.4903]	0.0499	0.2233	26.14	73.2	.8765
	3	2	0.6259	[0.0000; 1.0000]	0.0326	0.1805	4.73	78.9	.2539

*Note*: Significant interactions (<.05) are bold in table 2A. *p*‐value under *p* = .05 are bold in table 2B.

#### 3.3.2 PO between methods

**FIGURE 5:**
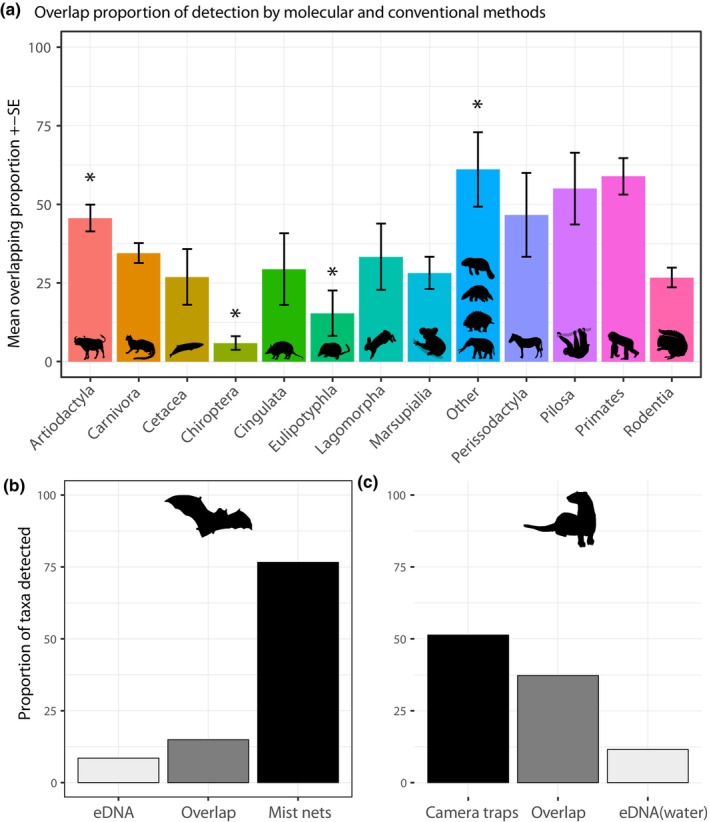
(a) Mean overlapping proportion of species detected by both methods (±SE) according to taxonomic order across all studies. * Represents significant variation in the effect sizes for each taxon according to moderator analysis. (b) Variation in the proportion of species detected according to detection methods for chiropters and (c) carnivores. Overlap stands for the proportion of taxa detected by both methods.

Variation in the estimates for PO between the two detection methods was significant according to taxonomy (*Q* = 120, *p* < .0001) with moderate heterogeneity (*I* ^2^ = 56.2%). Higher OPs between methods were observed for Artiodactyla (49%), while Chiroptera and Eulipotyphyla (<10%) were significantly less detected by both methods (Table 2B, Figure 5a). Null overlapping proportions are observed when animals are aerial, compared to other habitat type (0%, Table 2B). The type of barcoding sample also significantly influenced estimates PO (*Q* = 10.34, *p* = .035), with highly variable overlapping proportion when soil is used as DNA sample (42%, 95% CI = 35–48). In the same way, type of traditional methods also influenced overlapping proportion of taxa detected (*Q* = 30.5, *p* < .0001) with increasing proportion when researchers used camera traps. Greater overlapping detection proportions are also significantly found if a custom database is used for molecular taxonomical identification (*Q* = 21.8, *p* < .0001, Table 2B), while the number of used barcodes did not significantly correlate with OP estimates (*Q* = 5.2, *p* = .076).

### 3.4 Meta‐regression analysis

#### 3.4.1 LRR – taxa only detected by methods

In the multivariate analysis, 58.7% of the total heterogeneity in the dataset was accounted for (*F* = 2.0156, *p* < .0001, *I* ^2^ = 7%). The interactions of both types of molecular and traditional detection methods as well as taxonomy of the species impacted LRR (Table 3). Strikingly, using mist nests to detect chiropters allowed significantly more taxa than eDNA (Figure 5b), as well as camera traps for carnivores and marsupials. Otherwise, eDNA extracted from water samples allows an increased rodents, marsupials and cetaceans detection compared to surveys and camera traps (Table 3; Figure 5c).

**TABLE 3:** Log risk ratio meta‐regression analysis results for significant interactions.

Log risk ratio			Estimate	SE	*T*‐value	df	*p*‐Value	CI lower bound	CI upper bound
Intercept			−0.0885	0.2457	−0.3603	242	0.7189	−0.5726	0.3955
Barcode sample type	Traditional method	Taxa							
Water	Camera traps	Carnivora	0.5047	0.2706	1.8655	242	0.0633	−0.0282	1.0377
**Water**	**Trawl surveys**	**Cetacea**	**−2.8559**	**1.3032**	**−2.1914**	**242**	**0.0294**	**−5.4230**	**−0.2888**
**iDNA**	**Camera traps**	**Chiroptera**	**−1.8886**	**0.6568**	**−2.8753**	**242**	**0.0044**	**−3.1824**	**−0.5947**
iDNA	Field surveys and transects	Chiroptera	−1.5209	0.8682	−1.7519	242	0.0811	−3.2310	0.1892
iDNA	Mist nets	Chiroptera	1.1872	0.6615	1.7946	242	0.0740	−0.1159	2.4902
Soil	Camera traps	Chiroptera	−1.6828	0.8573	−1.9628	242	0.0508	−3.3716	0.0060
**Water**	**Camera traps**	**Chiroptera**	**−1.8008**	**0.7254**	**−2.4827**	**242**	**0.0137**	**−3.2297**	**−0.3720**
**Water**	**Field surveys and transects**	**Chiroptera**	**−1.7224**	**0.7015**	**−2.4552**	**242**	**0.0148**	**−3.1043**	**−0.3405**
**Water**	**Mist nets**	**Chiroptera**	**1.5022**	**0.3953**	**3.8000**	**242**	**0.0002**	**0.7235**	**2.2809**
Soil	Camera traps	Marsupialia	0.6313	0.3630	1.7394	242	0.0832	−0.0836	1.3463
**Water**	**Field surveys and transects**	**Marsupialia**	**−1.5787**	**0.6358**	**−2.4829**	**242**	**0.0137**	**−2.8312**	**−0.3262**
**Water**	**Field surveys and transects**	**Rodentia**	**−0.7105**	**0.3407**	**−2.0853**	**242**	**0.0381**	**−1.3817**	**−0.0393**

*Note*: Significant interactions ( < .05) are in bold. Estimates in blue translate a higher log‐ratio risk, corresponding to more detection from traditional survey methods compared to eDNA, while estimates in pink translate more detection from eDNA compared to traditionals methods.

#### 3.4.2 PO between methods

A meta‐regression model for the OP of taxa detection accounted for 67.9% of the total variation within the dataset and moderator differences in OP were statistically significant (*F* = 2.0998, *p* < .0001; *I* ^2^ = 13.4%). Greater overlapping proportion between methods was in majority directed towards large taxa such as Primates, Perissodactyla, Artiodactyla, and Carnivores (Table 4).

**TABLE 4:** OP Meta‐regression analysis results for significant interactions.

Overlap proportion			Estimate	SE	*t*‐Value	df	*p*‐Value	CI lower bound	CI upper bound
Intercept			0.2759	0.1510	1.8269	242	0.0689	−0.0216	0.5734
Barcode sample type	Traditional method	Taxa							
Soil	Camera traps	Artiodactyla	0.9022	0.3246	2.7796	242	0.0059	0.2628	1.5416
Water	Camera traps	Artiodactyla	0.9022	0.3246	2.7796	242	0.0059	0.2628	1.5416
iDNA	Camera traps	Artiodactyla	0.4794	0.1719	2.7881	242	0.0057	0.1407	0.8181
Water	Field surveys and transects	Artiodactyla	0.4067	0.1964	2.0711	242	0.0394	0.0199	0.7936
Soil	Camera traps	Carnivora	0.5413	0.2201	2.4589	242	0.0146	0.1077	0.9750
Water	Field surveys and transects	Carnivora	0.3917	0.1751	2.2375	242	0.0262	0.0469	0.7366
Water	Camera traps	Carnivora	0.3843	0.1627	2.3614	242	0.0190	0.0637	0.7048
iDNA	Camera traps	Eulipotyphla	0.9022	0.4335	2.0813	242	0.0385	0.0483	1.7561
Water	Camera traps	Eulipotyphla	0.6609	0.2940	2.2478	242	0.0255	0.0817	1.2401
Soil	Camera traps	Lagomorpha	0.7411	0.2746	2.6989	242	0.0074	0.2002	1.2821
Soil	Camera traps	Marsupialia	0.5095	0.2144	2.3760	242	0.0183	0.0871	0.9319
Water	Cage trapping	Marsupialia	0.4656	0.2207	2.1094	242	0.0359	0.0308	0.9004
Water	Camera traps	Other	0.9022	0.3246	2.7796	242	0.0059	0.2628	1.5416
Soil	Camera traps	Other	0.9022	0.4335	2.0813	242	0.0385	0.0483	1.7561
iDNA	Camera traps	Other	0.5590	0.1994	2.8030	242	0.0055	0.1662	0.9518
Water	Field surveys and transects	Perissodactyla	0.9022	0.4335	2.0813	242	0.0385	0.0483	1.7561
Water	Camera traps	Perissodactyla	0.6960	0.2225	3.1288	242	0.0020	0.2578	1.1342
iDNA	Field surveys and transects	Pilosa	0.9544	0.2940	3.2461	242	0.0013	0.3752	1.5336
Water	Camera traps	Pilosa	0.9022	0.3246	2.7796	242	0.0059	0.2628	1.5416
Water	Field surveys and transects	Pilosa	0.6317	0.2504	2.5226	242	0.0123	0.1384	1.1249
iDNA	Camera traps	Pilosa	0.6002	0.2424	2.4765	242	0.0140	0.1228	1.0776
Water	Field surveys and transects	Primates	0.8625	0.2373	3.6345	242	0.0003	0.3951	1.3300
iDNA	Field surveys and transects	Primates	0.7801	0.2504	3.1153	242	0.0021	0.2868	1.2733
iDNA	Camera traps	Primates	0.6208	0.1700	3.6511	242	0.0003	0.2859	0.9557
iDNA	Field surveys and transects	Rodentia	0.6563	0.2337	2.8079	242	0.0054	0.1959	1.1167
iDNA	Camera traps	Rodentia	0.3538	0.1676	2.1111	242	0.0358	0.0237	0.6839
Water	Camera traps	Rodentia	0.3280	0.1645	1.9939	242	0.0473	0.0040	0.6521

## 4 DISCUSSION

An important consideration in interpreting our results is that data reviewed in this analysis are not a random sampling. We recognize that each study would have its own questions, motivations, and limitations for comparing survey methods which introduce inherent biases of these results. Moreover, the fact that only 28 articles matched the inclusion criteria shows the lack of replication for this type of comparative study in mammals, compared to other taxa present in aquatic ecosystems (Carvalho et al., 2021; Keck et al., 2022). This does, however, provide us the opportunity to identify these biases and report trends in the literature which will be crucial to evaluate which techniques allow greater species detection.

Overall, this study shows that detection success is taxonomy dependent, whether it concerns conventional or DNA‐based methods. As stated by Beng and Corlett (2020), in order to enhance the detection probability, collecting biological samples should be done where the target is most likely to be detected, based on data ecology. So, it is very unlikely that one method fits all mammals, due to their extensive variation in size, physiology, behavior and ecology. In this way, it is not surprising that we found that traditional survey methods targeting specific taxa outperformed metabarcoding, especially mist nets for Chiroptera.

Despite the results obtained through our analysis, we need to recognize that data extracted from the included articles are highly biased towards camera trapping as a conventional method. Hence, we need more replication studies with other survey tools to confirm our results. However, it is logical that camera traps are a useful tool to survey carnivores due to their size and behavior (Seeber & Epp, 2022). However, because such methods are taxa targeted and that have been used and perfected for decades, it is important to acknowledge the promising future of DNA metabarcoding and that sampling methods still need standardization (Thomsen & Willerslev, 2015). All articles used the same barcodes (COI, 16S, and 12S) with almost the same primers. They also used next‐generation Illumina sequencing and most of them used the same public reference databases (NCBI, EMBL, BOLD). However, custom databases yielded greater overlapping proportions of taxa detected, showing the importance of knowledge and reference barcodes of the area of interest. Moreover, it is critical to notice that sampling collection varied between studies in the number of samples, space, and time, greatly influencing taxa detection. This sampling variation is observed in our results through high between studies heterogeneity values (*I* ^2^) in pairwise moderator analysis. This shows that despite unaccounted indicator variation for moderators, we do find differences between moderators overall.

The use of iDNA for mammal inventory also seemed promising due to the facility to trap invertebrates and the DNA quality coming from blood as a substrate. However, our analysis showed that this method still needs improvements. As stated by Calvignac‐Spencer et al. (2013), the host preferences of the blood‐feeding invertebrates are still poorly understood, as blood meals might not reflect local host availability in density, space, and time. Overall, this method needs further studies to investigate host range variation, as it would be expected to be taxa‐specific and no one invertebrate fits all.

eDNA, however, had strong detection overlaps with conventional methods for specific taxa such as ungulates and primates. This evidences that it can be an adequate method for specific uses. All surrounding over 50% of detection overlap between methods, one however does not outperform the other, but rather complements each other. Given the practicality of eDNA sampling, it is also necessary to acknowledge that conventional methods also face challenges, especially because they are time‐consuming and require a lot of manpower. Most of all, they rely on taxonomic expertise which is becoming scarce (Hopkins & Freckleton, 2002; Wägele et al., 2011). However, we argue that even if taxonomists are not needed for eDNA metabarcoding sampling, they still have a crucial role, as well as their natural collections, to provide accurate identification based on barcode sequences coming from reference holotypes (De Santana et al., 2021; Paknia et al., 2015). On the way around, low detection overlaps highlight the need to use both types of methods to increase the overall success of mammalian inventories, and this is particularly the case for bats (Figure 2). The use of air filter to gather bat DNA is promising and could greatly complement mist netting (Garrett et al., 2023; Johnson et al., 2023). In the same way, the ease of sampling and high DNA yield from leaf swabs seems a promising technique for eDNA capture (Lynggaard et al., 2023), which predicts a bright future for eDNA biodiversity assessments.

Here, we want to argue that our results show that eDNA metabarcoding still needs conventional methods for cross‐validation and to reduce the chance for not detecting a species. Sampling strategy for biodiversity assessment should not be overlooked and thought based on the environment's locality, as well as the targeted taxa. Moreover, using both molecular and traditional tools can be combined to expand detection success. First, in a way that one can increase the chances to find mammalian DNA by increasing the diversity of substrates (iDNA, water, soil, vegetation, feces…) that makes sense in light of the targeted ecology's DNA (Barnes & Turner, 2015). Second, if a particular taxon is targeted, a combination of both DNA and trapping can maximize detection. For example, airDNA has been shown to be a promising way of detecting bat species (Garrett et al., 2023), which is completely noninvasive, and coupled with mist net surveys, to cross‐validate metabarcoding results. The “tangible proofs” coming from direct observations of specimens is still important because it is not possible to ignore the presence of eDNA in the absence of living target and the absence of eDNA in the presence of the living target without actual field surveys (Beng & Corlett, 2020). Another example of method combination for carnivores can be to use DNA traps to obtain hair and saliva from baits, as well as camera trapping. As taxonomic expertise and field experience are crucial to apprehend the challenges that one can face when sampling a location for animal detection, a greatly beneficial partnership between taxonomists, naturalists and molecular biologists has to be developed. This would also help in the implementation and enrichment of local/international reference databases, that can lack in under‐sampled locations or environments.

We conclude that when it comes to biodiversity assessment, especially for mammals, there is no one size fits all, and it is up to biologists to find the appropriate threshold between molecular tools and conventional survey methods to maximize detection success. However, eDNA still being at its infancy compared to traditional survey methods, this study shows that it is a promising and powerful tool. As already discussed by Carvhalo et al. (2022), there is urgency to conduct more comparison studies between methods, as well as a need to better understand the ecology of the DNA targeted. This will allow us to further tune methods for the most accurate biodiversity assessment.

## AUTHOR CONTRIBUTIONS

**Pauline Van Leeuwen:** Conceptualization (equal); data curation (equal); formal analysis (equal); investigation (equal); methodology (equal); project administration (equal); software (equal); visualization (equal); writing – original draft (equal). **Johan Michaux:** Conceptualization (equal); funding acquisition (equal); project administration (equal); resources (equal); supervision (equal); validation (equal); writing – review and editing (equal).

## CONFLICT OF INTEREST STATEMENT

The authors declare that there are no conflicts of interest.

## Supporting information


Appendix S1

## Data Availability

Data and R script are available from the Figshare Repository: https://figshare.com/articles/dataset/Meta‐analysis_eDNA_vs_conventional_surveys/23284298.
